# Urbanization enhances ornament expression in a common waterbird

**DOI:** 10.1093/beheco/araf056

**Published:** 2025-05-24

**Authors:** Amelia Chyb, Radosław Włodarczyk, Jan Jedlikowski, Piotr Minias

**Affiliations:** University of Lodz, Faculty of Biology and Environmental Protection, Department of Biodiversity Studies and Bioeducation, Banacha 1/3, 90-237, Lodz, Poland; University of Lodz, Faculty of Biology and Environmental Protection, Department of Biodiversity Studies and Bioeducation, Banacha 1/3, 90-237, Lodz, Poland; Faculty of Biology, Biological and Chemical Research Centre, University of Warsaw, Żwirki i Wigury 101, 02-089, Warsaw, Poland; University of Lodz, Faculty of Biology and Environmental Protection, Department of Biodiversity Studies and Bioeducation, Banacha 1/3, 90-237, Lodz, Poland

**Keywords:** birds, condition, ornaments, the Eurasian coot, urbanization

## Abstract

In birds, many components of visual communication (ie plumage and non-plumage ornaments) play an important role in signaling of individual quality. It is widely acknowledged that ornament expression may be modulated by environmental conditions, however, it remains relatively poorly explored whether and how urbanization affects the expression of non-plumage ornamentation in urban dwelling-individuals. Here, we investigated the effect of urbanization on the expression of bare-part (non-plumage) putative ornament (ie the frontal shield size) across eight populations of a common reed-nesting waterbird, the Eurasian coot *Fulica atra*. Most importantly, we found robust support for the positive effect of urbanization on shield size and its condition-dependent character in coots. Also, long-term monitoring of a single urban population revealed relationships between the ornament expression and nest site selection patterns, as coots with larger shields were bold enough to colonize more anthropogenically transformed urban sites with stronger human disturbance and better accessibility to anthropogenic food. At the same time, we found no support for associations between the shield size and either nest defense behavior or reproductive performance in coots, likely reflecting stochasticity of anthropogenic selective pressures. To our knowledge, this study provides the first evidence for the enhanced expression of an unpigmented non-plumage putative ornament in urban-dwelling birds. Our results show that the effects of urbanization on non-plumage components of quality signaling in birds may be complex and multifaceted, and reinforces a need for further investigation focusing on different types of ornamentation across divergent avian species.

## Teaser

It is well documented that birds use various ornaments to advertise their quality to potential sexual partners or competitors. However, what makes a significant impact on the strength of this message? Our study shows that condition-dependent expression of avian ornamentation is higher in urban environments, as urban Eurasian coots had significantly bigger frontal shields than nonurban ones, and these differences were primarily attributed to variation in body condition.

## Introduction

In recent decades, human population growth has led to significant alterations in land use and promoted rapid urban spatial expansion (McNeill 2006). According to the United Nations (2019), the percentage of the human population living in cities exceeded the percentage of people living in rural areas in 2007 for the first time in history. The expansion of urban residential areas, land transformation into agricultural grounds, deforestation, and intensive development of transport networks result in habitat loss and fragmentation of the natural environment (Scanes 2018). Limited availability of natural living space can lead to the reduction of animal populations, species extinction, and global loss of wildlife biodiversity (Hanski 2011). Human-dominated areas generate multiple stressors (eg elevated human disturbance, pollution, noise, and artificial light), which usually occur in the natural environment to a very limited extent or not at all (Isaksson 2018). Some bird species show higher tolerance for urban environmental conditions than others and establish stable breeding populations in human-dominated areas (so-called urban adapters; Croci et al. 2008). Individuals from these urban populations often show remarkable phenotypic differences compared to individuals from wildland, resulting from physiological, morphological, and behavioral adaptation (eg Seress and Liker 2015; Isaksson 2018).

Urbanization can markedly affect body condition in birds via synergistic action of multiple biotic and abiotic environmental factors, such as increased availability of anthropogenic food sources, high population density, milder microclimate, elevated inter- and intraspecific competition for limited natural resources, and altered predator and parasite pressure (Seress and Liker 2015; Isaksson 2018). The complexity of bottom-up and top-down processes shaping the relationship between body condition and urbanization is also reflected by mixed results of ecological studies. For instance, urbanization enhanced several components of body condition in non-passerine (Auman et al. 2008) and passerine (Fokidis et al. 2008; Liker et al. 2008) birds. At the same time, decreased body condition was associated with a higher level of landscape urbanization in several other passerines (Meillère et al. 2015; Phillips et al. 2018; Grade et al. 2021; Peneaux et al. 2021). Some studies failed to reveal any differences in condition between urban and nonurban birds (Bókony et al. 2012) or showed contrasting effects of urbanization on different condition parameters (Powell et al. 2013). Although the complex impact of urbanization on body condition in birds is relatively well documented, there is little information on whether and how urbanization-related variation in body condition affects visual components of quality-signaling in urban-dwelling individuals.

In birds, exaggerated secondary sexual traits (ie sexual ornaments) such as colorful feather plumes, colouration of uncovered body parts, and structural appendages (eg wattles, spurs) play a significant role in sexual communication (Møller and Pomiankowski 1993). As the costs of ornament development and maintenance may include investments in substrates and energy, the ornament expression is generally acknowledged to be condition-dependent (eg Hegyi et al. 2020; Hernández et al. 2021; Nolazco et al. 2022). Thus, it can be expected that environmental conditions of urban habitats may significantly affect the expression of different components of quality-signaling via modulation of trophic structure (eg high and low availability of anthropogenic and natural food, respectively) and resulting alterations in body condition (Eeva et al. 1998; Cronin et al. 2022; Janas et al. 2024). Despite this expectation, empirical evidence for the effects of urbanization on ornament expression in wild-living birds is scarce, and it is mostly limited to a narrow representation of ornamentation types, primarily focusing on the carotenoid-based plumage coloration. For instance, the expression of carotenoid-based feather coloration decreases across an urbanization gradient in the great tit *Parus major* and the house finch *Haemorhous mexicanus* (Hõrak et al. 2000, 2001; Isaksson et al. 2005; Giraudeau et al. 2018; Sykes et al. 2021). In contrast, studies conducted in the northern cardinal *Cardinalis cardinalis* revealed either positive (Baldassarre et al. 2022) or negative (Jones et al. 2010) associations between the expression of carotenoid-based components of sexual signaling and the level of landscape urbanization, suggesting that local environmental conditions may play an important role in shaping ornament expression in birds. Specifically, urbanization enhanced the redness of bill in male northern cardinals, however, this study focused exclusively on the carotenoid-base coloration, but not on the morphological characteristics of the ornament, such as size or shape (Baldassarre et al. 2022). It has been also shown that urban conditions may show contrasting effects on different types of sexual ornaments, as anthropogenic pollution decreased carotenoid-, but not melanin-based feather coloration in the great tit (Grunst et al. 2020). Finally, there is evidence for the complex effects of urbanization on the visual components of quality-signaling in non-avian vertebrates. For instance, male western fence lizards *Sceloporus occidentalis* had larger colorful throat patches than nonurban ones, however, the size of the ornament was associated with probability of ectoparasite infection, but not with condition and corticosterone level (Putman et al. 2025). Taking all this into account, expanding urban ecology research to other types of ornamentation (eg non-plumage structural ornaments) is likely to provide novel insights into how urbanization modulates the processes of visual communication in wildlife.

Many bird species from the Rallidae family have evolved a special ornamental structure on the head (so-called frontal shield), which is thought to signal condition, dominance, and competitive ability, thus playing a putative role in the assessment of potential mates and competitors (Crowley and Magrath 2004; Alvarez et al. 2005; Dey et al. 2014). In this study, we sought to determine whether unpigmented non-plumage ornamental structure (ie shield) functions as a signal (or ornament) in a common rallid waterbird, the Eurasian coot *Fulica atra*. For this purpose, we used data from 12-yr monitoring of a single urban population to test for associations of frontal shield size with behavior, nest-site selection, and reproductive performance. Next, to quantify the effect of urbanization on shield size, we combined extensive across-population sampling and long-term population monitoring approaches. In total, the shield size and body condition were measured and compared between ca. 480 coots from four urban and four nonurban populations in Central Europe (Poland). We hypothesized that shield size may be larger in urban than nonurban coots, driven by elevated body condition, as urban coots have previously been shown to readily exploit anthropogenic food resources, which may positively affect their nutritional status (Minias et al. 2018). Also, the physiological condition of coots has already been shown to increase along the urbanization gradient, from suburban to urban zones (Minias 2016). The size of frontal shield in rallids may be regulated hormonally and correlate positively with testosterone level (Gullion 1951; Eens et al. 2000). Therefore, we expected that better condition of urban coots may allow them to effectively bear the indirect costs of larger shields and elevated testosterone levels, such as higher energy expenditure and stronger engagement in aggressive social interactions. We also hypothesized that, due to higher level of testosterone, coots with larger shields may be bolder and less responsive to anthropogenic stress. Specifically, we expected that they would more often select nest sites exposed to higher human disturbance (but providing better access to anthropogenic food), show stronger nest defence behaviors, and show lower levels of physiological stress markers (heterophil/lymphocyte ratios). Finally, we expected that stronger expression of frontal shield size in coots should positively correlate with their reproductive performance (ie hatching and breeding success).

## Material and methods

### Study area and general field procedures

**Figure 1.**
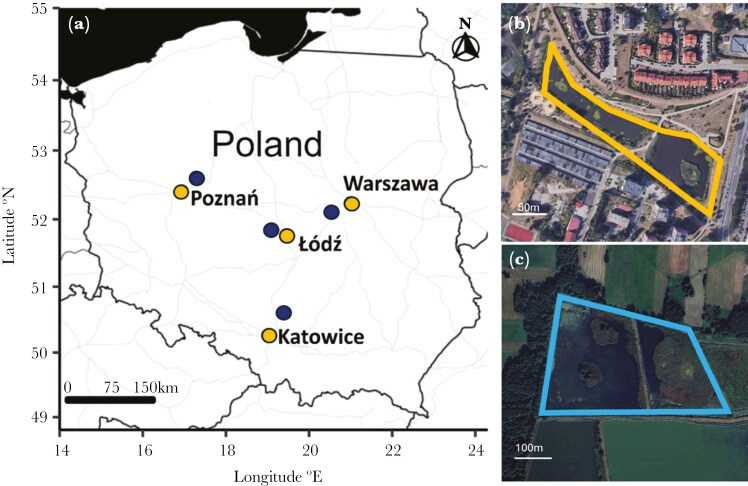
Location of urban (yellow) and nonurban (dark blue) sampling sites of the Eurasian coot (A). Satellite views show examples of differences in landscape urbanization and the share of reed vegetation cover in urban (B) and nonurban (C) sampling sites (the borders of waterbodies were highlighted). Satellite map data © 2021 Google, CNES/Airbus, MGGP Aero, Maxar technologies.

The Eurasian coots were sampled during the breeding season (March—early September) from 2012 to 2023 across four urban and four nonurban populations in Poland (Fig. 1). Urban populations were located in four city agglomerations (each over 650,000 inhabitants and over 290 km^2^ of surface): Warszawa (52°13′48″ N, 21°00′40″ E), Poznań (52° 24′ 30” N, 16° 56′ 01” E), Katowice (50° 15′ 30” N, 19° 01′ 39” E), and Łódź (51° 46′ 37” N, 19° 27′ 17” E). Urban sampling sites were located in densely urbanized areas, mostly at artificial reservoirs characterized by poorly developed emergent vegetation (<5% of water surface covered with reeds), high level of noise and artificial light, traffic-related pollution, elevated human activity, and high availability of anthropogenic food sources. Each urban population was paired with a corresponding nearby nonurban population located < 40 km away (Fig. 1). Nonurban sampling sites were located at fishponds and natural waterbodies surrounded mostly by natural and semi-natural landscape, including agricultural grounds, wastelands, and forests. All nonurban sites were characterized by rich emergent vegetation (>50% water surface covered with reeds), low level of noise and artificial light, and limited human activity.

In total, we caught 485 coots, usually 28 to 70 individuals per population, except for the Łódź urban population (197 individuals), which was extensively monitored throughout the entire study period. Because of this long-term monitoring, we also recaptured 39 individuals from Łódź across different years (breeding seasons). Most coots were captured with noose traps made from braided fishing line placed on the nest during incubation. Some individuals were caught while feeding on the shore or directly by hand at the nest (only at urban sampling sites). Captured birds were marked with a metal ring (left tarsus) and white plastic neck collar with a unique alphanumeric code to avoid unintentional recaptures of the same individuals. Each bird was weighed with an electronic balance (±1 g), and we collected basic morphological measurements, including wing length (± 1 mm).

Ca. 10 µl of blood from the tarsus vein was collected from each bird to assess the level of hemoglobin concentration (body condition) and leukocyte profiles (physiological stress) (see below). An additional 50 µl of blood was collected in 96% ethanol for the purpose of molecular sexing. Genomic DNA was extracted using a DNA Purification Kit (Euryx, Gdańsk, Poland), and the sex of each bird was determined following the protocol developed by Griffiths et al. (1998).

### Frontal shield size

**Figure 2.**
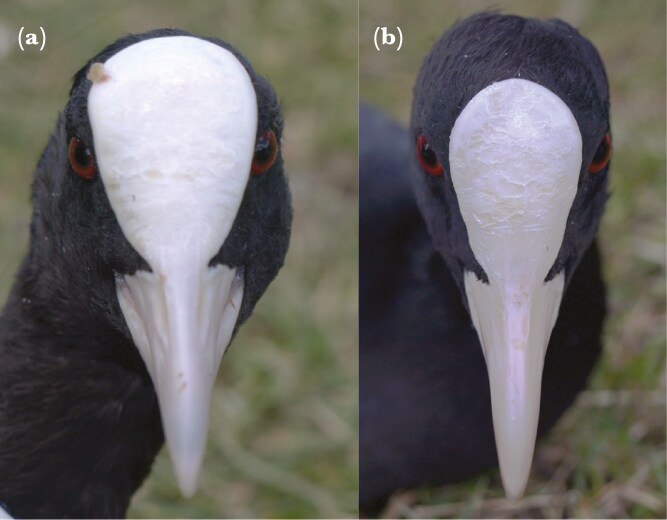
Observed variation in the frontal shield size of the Eurasian coot with an example of large shield (A) and small shield (B).

In the Eurasian coot, both males and females have a white fleshy frontal shield protruding above the skin surface and extending from the base of the bill up to the forehead end (Snow et al. 1997). Previous studies revealed that the frontal shield in coots is sexually dimorphic (larger shields in males; Minias 2015). Here, the frontal shield size (Fig. 2) was used as a proxy of putative ornament expression. Due to the considerable variation in the shape of the frontal shield in coots, it was technically unfeasible to effectively measure its actual area during the fieldwork. Therefore, two linear measurements were made with the calipers to determine shield size (both ± 0.1mm): length (in the midline from the highest point of the shield to the base of the beak) and width (at the widest point of the shield). The mean shield length was 28.73 ± 0.14 (ranged from 18.4 to 40.6mm) and the mean shield width was 19.57 ± 0.12 (ranged from 13.3 to 29.2mm). To characterize shield size, both measurements (length and width) were combined into a single univariate parameter (PC1) using principal component analysis (PCA). Although length and width of the shield were relatively well correlated (Pearson product-moment correlation coefficient: r = 0.81, P < 0.001), a combination of the two correlated measurements was likely to better reflect the total size (area) of the shield than a single linear measurement. In fact, PC1 accounted for 91% of the variability in length and width of the frontal shield, while one measurement reflected only 65% (R^2^ = 0.65) of variance in the second measurement. To eliminate sex-related variation in the frontal shield size, PC1 was standardized to z scores separately for males and females. To assess within-individual repeatability of shield size between years (breeding seasons), repeated measurements of frontal shield size were collected from 39 recaptured individuals (Łódź urban population). If the individual was recaptured in more than 1 yr (breeding season), only the measurements collected during the first recapture were used in the calculation of repeatability.

### Condition indices

We used two parameters to assess the body condition of coots: total blood hemoglobin concentration and size-corrected body mass (scaled mass index, SMI). In birds, hemoglobin concentration is considered a robust indicator of physiological body condition that reflects the oxygen-carrying capacity of blood and correlates with diet quality, health, and fitness (reviewed in Minias 2015a). Here, total blood hemoglobin concentration was measured using a modified azide methemoglobin reaction with a portable HemoCue Hb 201 + photometer (HemoCue, Ängeholm, Sweden). In contrast, SMI is commonly used as a proxy of energy stores in birds (Jacobs et al. 2012; Nip et al. 2018) and, here, it was calculated using an equation developed by Peig and Green (2009):


$$\begin{array}{l} SMI=M_{i}{\left[\frac{L_{0}}{L_{i}}\right]}^{b_{SMA}} \end{array}$$

where M_i_ - individual body mass, L_i_ - individual wing length as a linear body measurement, L_0_ - mean sex-specific wing length, and b_SMA_ - scaling exponent estimated by the standardized major axis regression of M on L. SMI was calculated separately for males and females. We collected 481 hemoglobin concentration and 522 SMI measurements from 425 and 467 individuals, respectively.

### Physiological stress

We used leukocyte profiles to assess the physiological responses of coots to urban-related stressors. For this purpose, smears were prepared from fresh blood, dried in the air, coloured with the May-Grünewald-Giemsa method, and examined under the microscope using 1000x magnification with immersion oil. In each blood smear, 100 white blood cells (WBC) were randomly selected and assigned to five categories: heterophils (H), lymphocytes (L), basophils (B), eosinophils (E), and monocytes (M). For each bird, we calculated the H/L ratio (ie the proportion of heterophil to lymphocyte number), which is considered a robust indicator of physiological stress in wild-living birds (Johnstone et al. 2012). A wide range of ornithological studies showed that variant stressors (eg parasitic infections, pollution, noise) affect the proportion of WBC types circulating in blood, increasing the number of heterophils and decreasing the number of lymphocytes (eg Davis et al. 2008; Ribeiro et al. 2022). In total, we collected 479 measurements of H/L ratio from 430 individuals. The measurements were log-transformed due to strong right skewness (3.01).

### Long-term monitoring of the urban population

Cross-population comparisons of ornament expression were complemented with data from extensive long-term monitoring of a single urban coot population from Łódź. In this population, we quantified nest site selection, behavior (nest defence), and reproductive performance of coots, which were captured for shield measurements. Laying date (ie the date of laying the first egg) was inferred from the observations of birds at the pre-laying stage and a single visit at the nest during the period of egg laying. Data on nest site selection and clutch size were collected during the second visit at the nest, usually 1 to 2 wk after incubation started. Additional visits at the nest were made to catch birds and collect behavioral data. All data were collected by two observers (PM and RW) across the entire study period (2012 to 2023). In total, we recorded 211 breeding attempts of captured birds, including 178 first and 33 second or renest (repeated after brood failure) broods.

#### Nest site selection.

**Figure 3.**
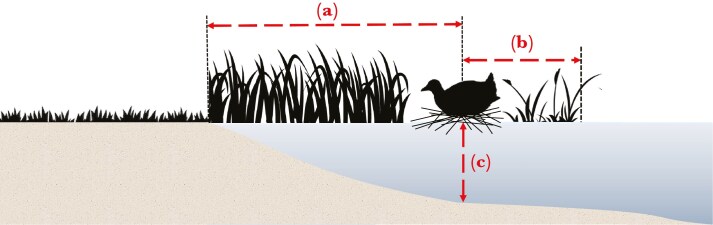
Nest site characteristics measured in Eurasian coots from an urban (Łódź) population: the distance from the nest to the shore (A), the distance from the nest to the open water (B), and the water depth at the nest (C).

Eurasian coots usually build nests in patches of dense emergent vegetation, which provides concealment against (mostly avian) predators (Snow et al. 1997). However, emergent vegetation is often limited or absent at artificial urban reservoirs, so effective colonization of urban areas by coots requires severe alterations in nest site selection patterns. In fact, some urban coots may nest in territories with no emergent vegetation, which, on the one hand, increases the risk of brood predation and may promote behavioral adaptations (eg elevated aggression and nest defence), but on the other hand, provides benefits resulting from human activity (eg access to rich anthropogenic food resources). Here, we measured three nest site characteristics: the distance from the nest to the open water (set to zero if the nest was located outside emergent vegetation), the distance from the nest to the shore, and the water depth at the nest (Fig. 3). In general, shorter distance from the nest to the shore and open water, and smaller water depth are characteristic of more transformed (urbanized) territories with less emergent vegetation. Nest site characteristics were recorded for 180 breeding attempts by 143 individuals. The distances from the nest to the shore and open water were log-transformed due to strong right skewness (1.43 and 2.15, respectively).

#### Nest defence behavior.

To assess the nest defence behavior of coots during an incubation stage, the experimenter approached each nest with an upright posture and at a moderate pace (see Knight and Temple 1986) and stood still directly next to the nest, observing the birds for 5 min (using binoculars if needed). Nest defence behavior was quantified as: (*i*) the presence or absence of aggressive nest defence (approach with threat posture, alarm vocalization, or splattering display) against a human intruder (experimenter) at the nest site (coded as 1 or 0, respectively), and (*ii*) approach distance, ie the minimum distance at which a bird approaches human intruder at the nest site. In the case of a direct bird’s attack with claws or beak on the intruder, the approach distance was set to zero. For each measurement, we recorded both the experiment date and the incubation stage (ie the interval between the laying date and the date of the experiment). Nest defence behavior was recorded for 128 individuals, but approach distance was measured only for 102 individuals that actively approached an experimenter at the nest. The approach distance was log-transformed due to strong right skewness (2.69).

#### Reproductive performance.

For each breeding attempt of coots, we measured five basic reproductive parameters: (*i*) laying date, (*ii*) clutch size, ie the number of laid eggs, (*iii*) hatching success, ie hatching of at least one chick in the brood or hatching no chicks (coded as 1 or 0, respectively), (*iv*) breeding success, ie fledging of at least one chick or no chicks (coded as 1 or 0, respectively), and (*v*) the number of raised chicks. Breeding success and the number of raised chicks were assessed visually (with binoculars) for all broods after 4 to 6 wk from hatching.

### Statistical analysis

Long-term monitoring data (Łódź urban population) were used to investigate associations of ornament expression with nest site selection, nest defence behavior, and reproductive performance, while cross-population data were used to assess the associations between putative ornament expression (frontal shield size) and urbanization level, body condition, and physiological stress (H/L ratio). All analyses were conducted with generalized linear mixed models (GLMMs).

First, we used long-term monitoring data to run GLMMs with nest site characteristics (distance from the nest to the shore, distance from the nest to open water, and water depth at the nest site), nest defence behavioral traits (presence of aggressive nest defence and approach distance), and reproductive parameters (laying date, clutch size, hatching success, breeding success, and the number of raised chicks) entered as response variables in separate models. In all these models, frontal shield size was included as a covariate. Laying date was included as an additional covariate in the analyses of nest site characteristics and all the remaining reproductive parameters, while the date of the experiment and incubation stage were used as covariates in the analyses of nest defence behavior. The status of the breeding attempt (first vs. second/renest brood) was used as a fixed factor in all these models, except for the analysis of laying date, which was limited to first broods. Sex was included as another fixed factor in the analysis of nest defence to control for behavioral differences between males and females. Next, to test for the cross-population relationships between ornament expression and urbanization, we ran GLMM with frontal shield size entered as a response variable, capture date included as a covariate, urbanization level as a fixed factor, and paired population identity as a random factor. To test whether associations between frontal shield size and urbanization can be explained by differences in body condition or physiological stress between urban and nonurban coots, we re-ran the same model with condition indices (hemoglobin concentration and SMI) and physiological stress (log H/L ratio) included as additional covariates. Finally, in all GLMMs (both cross- and within-population), wing length was entered as a covariate to control for variation in structural body size, while individual identity and year were entered as random factors to account for pseudoreplication resulting from repeated measurements of the same individuals and to control for annual variation in the measurements, respectively. To test whether the effects of covariates were similar across urban and nonurban landscape, we included interactions between the urbanization level and wing length, capture date, and condition parameters in the full cross-population models. All non-significant (p > 0.05) interactions were removed from the final models.

In all within-population models testing associations between the shield size and either nest site characteristics, behavioral responses, and reproductive performance (except for clutch size) data from both sexes were included. However, in most cases (92.4%), the measurements of shield size originated from only one partner per breeding attempt. In the remaining 7.6% of cases, we included the measurements from both partners in the models (n = 16 breeding attempts). To test whether including both partners in the dataset produced any pseudoreplication-related biases, we reran the models including only one partner per pair. The models yielded qualitatively similar results, providing support for no pseudoreplication issues.

All GLMMs were run in the statistical environment R v.4.2.3 (R Foundation for Statistical Computing, Vienna, Austria) using the *lme4* package (Bates et al. 2015), except the model for the number of chicks, which was run using the *glmmTMB* package (Brooks et al. 2017). Hatching success, breeding success, and the nest defence behavior (binary traits) were analyzed with a binomial distribution of the response variable, while the number of chicks was analyzed with a zero-inflated Poisson distribution. All other analyses were run with a Gaussian distribution of the response variable. To estimate the effect sizes of particular predictors in the GLMMs, we calculated semi-partial R^2^ statistics using the *r2beta* function from the *r2glmm* package applying the Nakagawa and Schielzeth approach (Nakagawa and Schielzeth 2013; Jaeger et al. 2017). Within-individual repeatability (R) in ornament expression (across years) was assessed with the interclass correlation coefficient, as implemented in the *irr* R package (Gamer et al. 2012). All values are reported as means ± SE.

## Results

**Figure 4.**
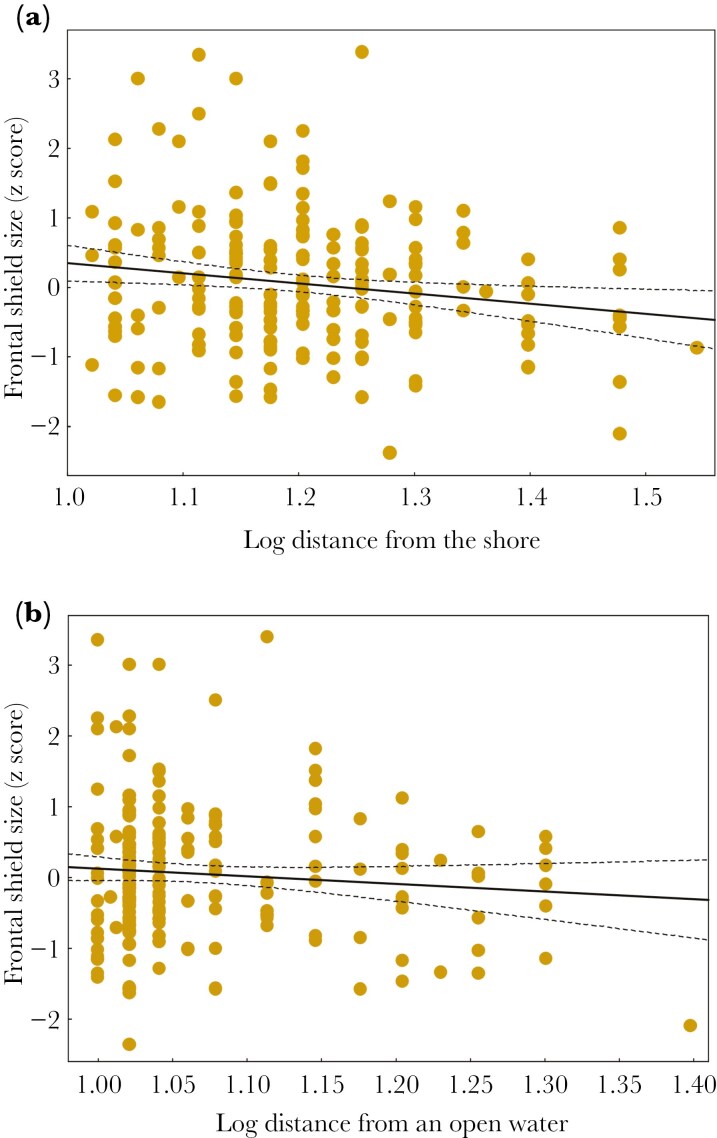
Associations of the frontal shield size with nest site characteristics: the distance from the nest to the shore (A) and distance from the nest to open water (B) in Łódź urban population of the Eurasian coot. Model details are presented in Table 1. Regression lines with 95% confidence intervals are shown.

The analysis of long-term monitoring data showed that frontal shield size was associated with nest site selection patterns in the urban population (Łódź). Individuals with larger shields built nests closer to the shore (β = −0.021 ± 0.008, P = 0.006, Table 1, Fig. 4) and closer to open water (β = −0.012 ± 0.006, P = 0.045, Table 1, Fig. 4) than individuals with smaller shields. The frontal shield size explained 3.9 % and 2.0% of the variance in the distance from the nest to the shore and open water, respectively (Table 1). We found no significant association between frontal shield size and water depth at the nest-site (P = 0.13, Table 1). We also failed to find any significant associations of frontal shield size with nest defence behavior (the presence of aggressive nest defence and approach distance) and reproductive parameters (laying date, clutch size, hatching success, breeding success, and the number of chicks) (all P > 0.05, Tables S1-S7). We found significant repeatability (R = 0.31, 95% Cl: 0.02 to 0.56; P = 0.018) in measurements of the frontal shield size across years.

**Table 1.** The results of the general linear mixed models testing for associations between ornament expression (frontal shield size) and nest site characteristics in Łódź urban population of the Eurasian coot. The year and individual identity were included as random factors. Significant predictors are marked in bold.

Nest site characteristic	Predictor	Estimate ± SE	df	t	P	R^2^
Distance from nest to the shore	**Intercept**	**0.671 ± 0.229**	**144.80**	**2.93**	**0.004**	**NA**
	**Frontal shield size**	**−0.021 ± 0.008**	**178.10**	**−2.77**	**0.006**	**0.039**
	**Wing length**	**0.003 ± 0.001**	**136.00**	**3.37**	**0.001**	**0.061**
	Breeding status	0.032 ± 0.023	165.70	1.37	0.17	0.009
	**Laying date**	**−0.0016 ± 0.0004**	**178.90**	**−3.49**	**0.001**	**0.061**
Distance from nest to open water	**Intercept**	**0.768 ± 0.169**	**151.00**	**4.55**	**<0.001**	**NA**
	**Frontal shield size**	**−0.012 **±** 0.006**	**172.30**	**−2.02**	**0.045**	**0.020**
	**Wing length**	**0.002 ± 0.001**	**144.00**	**2.27**	**0.025**	**0.026**
	**Breeding status**	**0.040 ± 0.018**	**183.90**	**2.25**	**0.026**	**0.023**
	Laying date	−0.0005 ± 0.0003	173.60	−1.52	0.13	0.011
Water depth	**Intercept**	**1.031 **±** 0.030**	**146.10**	**33.90**	**<0.001**	**NA**
	Frontal shield size	0.002 ± 0.001	168.30	1.52	0.13	0.012
	Wing length	0.0001 ± 0.0001	137.20	0.39	0.70	0.001
	Breeding status	−0.002 ± 0.003	189.70	−0.52	0.60	0.001
	Laying date	−0.0001 ± 0.0001	168.30	−1.29	0.20	0.009

**Figure 5.**
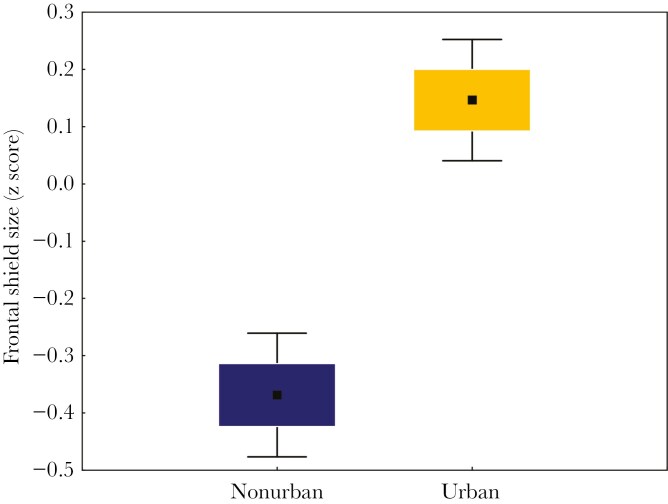
Differences in the frontal shield size between urban and nonurban populations of the Eurasian coot. Means (central points), SE (box), and 95% confidence intervals (whiskers) of the frontal shield size are shown.

**Figure 6.**
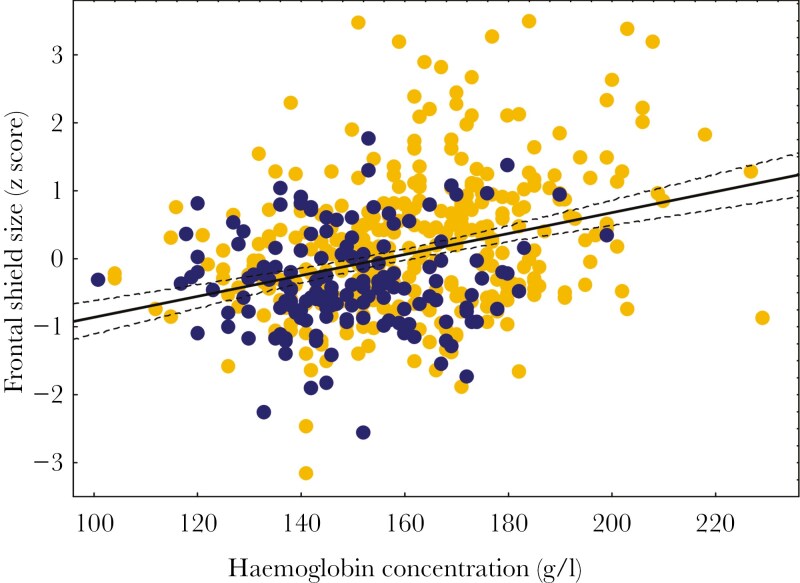
Association of the frontal shield size with total blood hemoglobin concentration in coots from urban (yellow) and nonurban (dark blue) populations. Model details are presented in Table 3. Regression line with 95% confidence intervals is shown.

The level of landscape urbanization was identified as a significant predictor of putative ornament expression (frontal shield size) across all populations (β = 0.321 ± 0.085, P < 0.001, Table 2, Fig. 5), and urban individuals had larger frontal shields compared to nonurban birds. We also found associations between frontal shield size and body condition, although this effect was not consistent across parameters. Specifically, a larger frontal shield was associated with a higher level of hemoglobin concentration (β = 0.005 ± 0.002, P = 0.026, Table 3, Fig. 6), but showed no significant relationship with either SMI (P = 0.49, Table 3) or physiological stress (H/L ratio; P = 0.65, Table 3). The landscape urbanization level remained significant after controlling for variation in body condition (β = 0.198 ± 0.099, P = 0.046, Table 3). In general, urbanization explained 2.9% of the variance in frontal shield size (PC1), but the effect decreased to 1.0% after controlling for body condition (Tables 2 and 3). At the same time, unstandardized mean differences in two raw measurements of shield size between urban and nonurban coots were 1.32 mm and 0.86 mm for female and male shield length, and 1.72 mm and 1.26 mm for female and male shield width. The standardized mean between-habitat difference was estimated at 5.7% across measurements and sexes. Finally, cross-population analyses revealed strong temporal variation in frontal shield size, as it decreased with date over the course of the breeding season (β = −0.014 ± 0.001, P < 0.001, Table 2). The capture date explained 17.7% and 14.9% of the variance in frontal shield size in the models without and with body condition, respectively (Tables 2 and 3). All interactions between urbanization level and other predictors were non-significant (all P > 0.05) and removed from the final models.

**Table 2.** The results of the general linear mixed model testing for associations between ornament expression (frontal shield size) and urbanization level across four urban and four nonurban populations of the Eurasian coot. The year and individual identity were included as random factors. Significant predictors are marked in bold.

Predictor	Estimate ± SE	df	t	P	R^2^
Intercept	−0.048 ± 0.963	492.75	−0.05	0.96	NA
**Urbanization**	**0.321 ± 0.085**	**469.18**	**3.79**	**<0.001**	**0.029**
**Capture date**	−**0.014 ± 0.001**	455.89	−10.33	**<0.001**	**0.177**
Wing length	0.008 ± 0.004	491.32	1.85	0.065	0.006

**Table 3.** The results of the general linear mixed model testing for associations between ornament expression (frontal shield size) and urbanization level after controlling for condition parameters (hemoglobin concentration and SMI) and physiological stress (H/L ratio) across four urban and four nonurban populations of the Eurasian coot. The year and individual identity were included as random factors. Significant predictors are marked in bold.

Predictor	Estimate ± SE	df	t	P	R^2^
Intercept	−0.476 ± 1.053	412.50	**−**0.45	0.65	NA
**Urbanization**	**0.198 ± 0.099**	**326.90**	**2.00**	**0.046**	**0.010**
**Capture date**	**−0.016 ± 0.002**	**402.60**	**−8.81**	**<0.001**	**0.149**
Wing length	0.006 ± 0.005	418.60	1.21	0.23	0.003
**Haemoglobin**	**0.005 ± 0.002**	**424.40**	**2.24**	**0.026**	**0.011**
SMI	0.0002 ± 0.0004	417.50	0.70	0.49	0.001
H/L ratio	0.155 ± 0.342	409.10	0.45	0.65	0.000

## Discussion

Our study provides correlative evidence that urbanization enhances the expression of the putative bare-part ornament in the Eurasian coot, as we found that coots from urban populations had significantly larger frontal shields than nonurban ones. We also found a positive association between the shield size and blood hemoglobin concentration, which stays in line with the condition-dependent nature of ornament expression in birds. Our analyses also revealed a significant relationship between putative ornament expression and two nest site selection characteristics, as urban coots with larger shields built less concealed nests located closer to the shore and open water. At the same time, we found no evidence for the correlations of shield size with either nest defence or reproductive performance, suggesting that shield size is not directly linked to aggressive behavior in coots and that individual quality (as signaled by ornament expression) may be a poor determinant of fitness (breeding success) in coots from an urban environment.

A growing body of evidence shows that ecological and environmental conditions of urban habitats can significantly affect visual components of quality-signaling in birds (Isaksson et al. 2005; Giraudeau et al. 2018; Sykes et al. 2021). However, our results are in stark contrast to the previous research, which has provided robust support for the detrimental effect of urbanization on the expression of carotenoid-based ornaments in passerines (so-called ‘urban dullness’ phenomenon; Salmón et al. 2023). While it has already been suggested that urbanization may differently affect avian ornaments arising via different physiological mechanisms (Grunst et al. 2020), so far there was little information on the impact of urbanization on non-plumage components of sexual signaling. Among the exceptions, urbanization has been shown to decrease the expression of carotenoid-based bare-part ornament in the common myna *Acridotheres tristis*, as urban individuals had less intensive eye skin colouration in comparison to nonurban individuals (Peneaux et al. 2021). In our study, we focused on the expression (size) of the unpigmented (white) non-plumage putative ornament in the Eurasian coot, which is likely determined via different physiological mechanisms than carotenoid-based colouration of feathers or skin. Our results provide evidence for the positive effects of urbanization on bare-part ornamentation in birds. This pattern was found to be robust and unlikely to reflect local effects, as it has been inferred based on the extensive sampling across four pairs of urban and nonurban coot populations.

The proportion of variation in the shield size explained by urbanization level was relatively small (1% to 3%), probably due to the dynamic changes in the shield size during the breeding season (ie the shield size decreases as the breeding season progresses), which increased variance within each habitat. In fact, the date explained the highest proportion of the variation in the shield size (14.9% to 17.7%). These results stay in line with a previous study by Visser (1988), which revealed marked temporal variation in the frontal shield size in a Dutch population of the Eurasian coot, with a clear peak at the beginning of the breeding season (March). On the other hand, we found that the two linear measurements of the frontal shield (length and width) were on average larger by 5.7% in urban than nonurban coots, and the relative difference in the total shield size area (being a product of both linear measurements) was likely to be even greater. It seems probable that the difference of this magnitude should be perceivable for birds and biologically relevant for quality signaling. At the same time, the taxon-specific character of association between urbanization and ornament expression cannot be excluded, which underpins the need for expanding future urban ecology research to different types of ornamentation across divergent avian species.

One of the most important characteristics of an urban environment is the high availability of anthropogenic food resources, which are usually willingly used by urban-dwelling birds (Isaksson 2018). Human-subsidized food (eg wheat bread, meal leftovers) is usually rich in carbohydrates and high-calorie, but at the same time, it may be deficient in essential nutrients, such as proteins (Murray et al. 2018; Burt et al. 2021). Therefore, a diet based on anthropogenic food supplies can effectively satisfy extensive caloric needs of urban individuals, but it may also cause some nutritional deficiencies, leading to a deterioration of certain physiological parameters (Murray et al. 2018; Bernat-Ponce et al. 2023; but see Auman et al. 2008). Different types of ornaments may respond differently to low micronutrient intake, depending on the physiological mechanism involved in their expression (Grunst et al. 2020). In birds, carotenoid compounds are not synthesized de novo in the organism, but have to be provided with food (Delhey et al. 2023). Therefore, the expression of carotenoid-based signals is often linked to the availability of high-quality natural food sources (eg plant seeds, caterpillars), which is usually limited in the human-dominated environment (Salmón et al. 2023). For instance, the narrow foraging niche was identified as a potential cause of decreased expression of carotenoid-based ornamentation in urban common mynas (Peneaux et al. 2021). In contrast, Baldassarre et al. (2022) suggested that enhanced expression of a carotenoid-based ornament (red bill and chest feathers in males and red underwing feathers in females) in urban northern cardinals was caused by diet supplementation with carotenoid-rich fruits of invasive plants. Here, we assume that the expression of the putative unpigmented fleshy ornament (frontal shield size) in coots may be more dependent on the total available energy reserves rather than on the nutritional quality of food. Our previous study showed that coots from our study urban populations are intensively fed by humans and willingly utilize anthropogenic food supplies (Minias et al. 2018), which can enhance their overall body condition.

Surplus energy reserves may also enable urban birds to maintain elevated levels of sex hormones, which is usually associated with severe physiological costs to the organism. For instance, the high level of testosterone is not only linked to increased energy expenditure via increased metabolic rate (Buchanan et al. 2001), but also to the suppression of crucial physiological functions, such as antioxidant activity and immunity (Alonso-Alvarez et al. 2007). The testosterone-dependent alterations in the frontal shield size were found in other rallid species, the common moorhen *Gallinula chloropus* and the American coot *Fulica americana* (Gullion 1951; Eens et al. 2000). High testosterone-dependent ornament expression may also result in significant social costs for birds via increased engagement in aggressive interactions with conspecifics (Webster et al. 2018). We assume that the Eurasian coots are likely to incur both physiological and social costs of elevated shield expression. Our results suggest that better condition (enhanced by urbanization) may allow to bear higher costs of ornament expression, regardless of their type. In fact, we found convincing support for the condition-dependent mechanism of putative ornament expression, as the size of the frontal shield positively correlated with the total blood hemoglobin concentration—a reliable proxy of physiological condition in birds (Minias 2015a). Hence, it seems likely that the effects of urbanization on ornament expression in coots are primarily mediated by variation in food availability across the gradient of urban and nonurban landscapes.

The expression of animal ornaments forms an inherent component of the complex network of mutual dependencies, including the activity of the hormonal system and behavior (Tibbetts 2014). Many studies show positive relationships between testosterone level and behavioral traits, such as aggression, boldness, and exploration (Wingfield and Ramenofsky 1985; Hau et al. 2000; Mougeot et al. 2005; Zysling et al. 2006; Raynaud and Schradin 2014; but see van Oers et al. 2011), as well as with ornament expression (Blas et al. 2006; Mougeot et al. 2005; Laucht et al. 2010). It seems probable that the expression of the frontal shield in coots may also be subject to hormonal regulation, thus having the potential to reliably signal variation in behavioral traits. In our study, coots with larger shield size selected less concealed nest sites located closer to the shore and open water, thus being more exposed to human disturbance and predation. This pattern was likely driven by greater boldness and exploratory behavior of coots with larger shields, allowing them to colonize more anthropogenically transformed sites within an urban landscape (eg the ones with little or no emergent vegetation). This scenario is also well consistent with the mechanism of testosterone-dependent expression of frontal shield size in rallid birds. However, the expression of shield size in coots may not only determine, but can also be modulated by nest site selection patterns. Nest sites more exposed to human disturbance are also characterized by a higher availability of anthropogenic food supplies, which may promote even stronger expression of the shield. Although the correlative character of our study does not allow us to clearly determine the cause-and-effect relationships between putative ornament expression and nest site selection in urban coots, it needs to be acknowledged that both mechanisms are not mutually exclusive. In fact, they may operate in a positive feedback loop, where bolder individuals with larger frontal shields colonize more transformed urban sites (consistent with testosterone-dependent ornament expression), which in turn allows them to take better advantage of anthropogenic food resources, thus further increasing frontal shield size (consistent with condition-dependent ornament expression). At the same time, we found no links between frontal shield size and nest defence behavior in coots, suggesting that expression of putative ornament is likely to better signal some behavioral traits (eg boldness) over others (eg aggression).

The long-term monitoring of a single urban population (Łódź) provided no evidence for the correlations of the frontal shield size with reproductive performance in the Eurasian coot. A wide range of studies conducted in natural nonurban habitats show, in general, positive associations of body condition and ornament expression with reproductive success in birds (eg Velando et al. 2001; Guindre-Parker et al. 2013; Hernández et al. 2021). However, environmental and ecological conditions in urban areas markedly differ from those prevailing in natural habitats, and this variation may significantly modulate the relationships between body condition, ornamentation, and reproductive performance. Birds nesting on urban waterbodies are often exposed to a broad spectrum of negative anthropogenic factors, such as elevated human disturbance (eg leisure activities), presence of non-natural predators (eg domesticated cats and dogs), high water level fluctuations, or strong eutrophication. We suggest that the absence of associations between shield size and reproductive performance in urban coots may be driven by the strong stochasticity and unpredictability of these anthropogenic processes, which can modulate bird reproductive output independently from individual quality. Our previous studies on the genetic variation of the Major Histocompatibility Complex (MHC), the key component of the adaptive immune system involved in pathogen recognition, revealed complex associations between MHC diversity and several fitness-related traits in urban coots (Pikus et al. 2022). Specifically, the MHC diversity was associated with ornament expression, body condition, laying date, and clutch size, but failed to correlate with reproductive output (Pikus et al. 2022). At the same time, anthropogenic eutrophication leading to harmful algal blooms in urban waterbodies was suggested as an important mechanism that reduced the breeding success of urban coots during periods of elevated temperatures (Chyb and Minias 2022). Taking all this into account, it seems plausible that under specific environmental conditions of human-dominated habitats, individual quality and body condition (as signaled by ornament expression) may play little role in shaping the reproductive output of urban waterbirds, such as the Eurasian coot.

In conclusion, our study provides evidence for the effects of urbanization on the expression of the putative unpigmented bare-part ornamental structure in birds, and it is one of the first to show a positive relationship between the urbanization level and ornament expression in urban-dwelling individuals. Our results are in stark contrast with the results of previous studies on carotenoid-based skin and plumage ornaments, which primarily focused on passerine bird species. Also, our results revealed the condition-dependent character of the ornament expression, suggesting that the frontal shield size may be considered as a putative signal of individual quality in the Eurasian coot, although the signaling value of this trait is not completely clear, especially in an explicitly sexual context (eg mate choice). Finally, we suggest that our results may reflect the stochastic nature of anthropogenic processes and their effect on fitness in urban birds, since despite condition-dependent expression, frontal shield size in coots did not translate into higher reproductive output. Our study shows that the effects of urbanization on non-plumage components of quality-signaling in birds may be complex and multifaceted, and reinforces a need for further investigation focusing on different types of ornamentation across divergent avian species.

## Supplementary Material

araf056_suppl_Supplementary_Tables_S1-S7

## Data Availability

Analyses reported in this article can be reproduced using the data provided by Chyb et al. (2025).
