# Supplementary material for: Urbanization enhances ornament expression in a common waterbird
Source: Behav Ecol. 2025 May 24;36(4):araf056. doi: 10.1093/beheco/araf056 (PMC12167231; doi:10.1093/beheco/araf056)
Supplement: araf056_suppl_Supplementary_Tables_S1-S7 [file araf056_suppl_supplementary_tables_s1-s7.pdf]

Supplementary_material_1

Urbanization enhances ornament expression in a common waterbird

Amelia Chyb[1], Radosław Włodarczyk[1], Jan Jedlikowski[2], Piotr Minias[1]

1. University of Lodz, Faculty of Biology and Environmental Protection, Department of Biodiversity Studies and Bioeducation, Banacha 1/3, 90-237, Lodz, Poland

2. Faculty of Biology, Biological and Chemical Research Centre, University of Warsaw, Żwirki i Wigury 101, 02-089, Warsaw, Poland

Correspondence and requests for materials should be addressed to: A.C. (email: chybamelia@gmail.com)

Table S1 The results of the general linear mixed model testing for associations between between ornament expression (frontal shield size) and the laying date in Łódź urban population of the Eurasian coot. The year and individual identity were included as random factors. Significant predictors are marked in bold.

Predictor	Estimate ± SE	df	t	P
**Intercept**	**191.625 ± 35.472**	**196.96**	**5.40**	**<0.001**
Frontal shield size	-0.918 ± 1.384	216.44	-0.66	0.51
**Wing length**	**-0.349 ± 0.165**	**196.11**	**-2.12**	**0.035**

Table S2 The results of the general linear mixed model testing for associations between ornament expression (frontal shield size) and the clutch size in Łódź urban population of the Eurasian coot. The year and individual identity were included as random factors. Significant predictors are marked in bold.

Predictor	Estimate ± SE	df	t	P
Intercept	4.183 ± 5.968	51.31	0.70	0.48
Frontal shield size	-0.244 ± 0.147	52.30	-1.66	0.10
Wing length	0.046 ± 0.026	43.15	1.75	0.087
Breeding status	-0.409 ± 0.425	80.85	-0.96	0.34
**Laying date**	**-0.047 ± 0.009**	**72.84**	**-5.45**	**<0.001**

Table S3 The results of the general linear mixed model testing for associations between ornament expression (frontal shield size) and the hatching success in Łódź urban population of the Eurasian coot. The year and individual identity were included as random factors.

Predictor	Estimate ± SE	t	P
Intercept	0.277 ± 4.576	0.06	0.95
Frontal shield size	0.054 ± 0.163	0.33	0.74
Wing length	0.009 ± 0.020	0.45	0.65
Breeding status	0.958 ± 0.680	1.41	0.16
Laying date	-0.010 ± 0.010	-1.01	0.31

Table S4 The results of the general linear mixed model testing for associations between ornament expression (frontal shield size) and the breeding success in Łódź urban population of the Eurasian coot. The year and individual identity were included as random factors. Significant predictors are marked in bold.

Predictor	Estimate ± SE	t	P
Intercept	2.320 ± 4.340	0.54	0.59
Frontal shield size	-0.172 ± 0.156	-1.10	0.27
Wing length	0.009 ± 0.019	0.45	0.65
**Breeding status**	**1.342 ± 0.588**	**2.28**	**0.022**
**Laying date**	**-0.033 ± 0.010**	**-3.21**	**0.001**

Table S5 The results of the general linear mixed model testing for associations between ornament expression (frontal shield size) and the number of raised chicks in Łódź urban population of the Eurasian coot. The year and individual identity were included as random factors. Significant predictors are marked in bold.

Predictor	Estimate ± SE	t	P
Intercept	1.087 ± 1.569	0.69	0.49
Frontal shield size	-0.098 ± 0.059	-1.67	0.095
Wing length	0.011 ± 0.007	1.62	0.11
Breeding status	0.381 ± 0.195	1.95	0.051
**Laying date**	**-0.020 ± 0.004**	**-4.85**	**<0.001**

Table S6 The results of the general linear mixed model testing for associations between ornament expression (frontal shield size) and the aggression in Łódź urban population of the Eurasian coot. The year and individual identity were included as random factors.

Predictor	Estimate ± SE	Z	P
Intercept	-10.309 ± 11.714	-0.88	0.38
Frontal shield size	-0.260 ± 0.269	-0.97	0.33
Wing length	0.070 ± 0.054	1.28	0.20
Breeding status	-0.344 ± 0.811	-0.43	0.67
Sex (M vs. F)	-0.592 ± 0.833	-0.71	0.48
Date of the experiment	-0.019 ± 0.018	-1.06	0.29
The incubation stage	-0.006 ± 0.035	-0.17	0.86

Table S7 The results of the general linear mixed model testing for associations between ornament expression (frontal shield size) and the approach distance in Łódź urban population of the Eurasian coot. The year and individual identity were included as random factors.

Predictor	Estimate ± SE	Df	t	P
Intercept	0.707 ± 0.462	145.40	1.53	0.13
Frontal shield size	0.0002 ± 0.0095	144.70	0.02	0.98
Wing length	0.002 ± 0.002	145.80	0.81	0.42
Breeding status	-0.009 ± 0.024	109.00	-0.38	0.71
Sex (M vs. F)	-0.038 ± 0.033	141.80	-1.13	0.26
Date of the experiment	0.0006 ± 0.0007	145.00	0.95	0.34
The incubation stage	-0.002 ± 0.001	102.70	-1.51	0.14
